# The branched receptor-binding complex of *Ackermannviridae* phages promotes adaptive host recognition

**DOI:** 10.1016/j.isci.2024.110813

**Published:** 2024-09-07

**Authors:** Anders Nørgaard Sørensen, Cedric Woudstra, Dorottya Kalmar, Jorien Poppeliers, Rob Lavigne, Martine Camilla Holst Sørensen, Lone Brøndsted

**Affiliations:** 1 Department of Veterinary and Animal Sciences, University of Copenhagen, Stigbøjlen 4, 1870 Frederiksberg C, Denmark; 2 Laboratory of Gene Technology, KU Leuven, Kasteelpark Arenberg 21 Box 2462, 3001 Heverlee, Belgium

**Keywords:** Virology, Cell biology

## Abstract

Bacteriophages can encode multiple receptor-binding proteins, allowing them to recognize diverse receptors for infecting different strains. *Ackermannviridae* phages recognize various polysaccharides as receptors by encoding multiple tail spike proteins (TSPs), forming a branched complex. We aimed to mimic the evolution of the TSP complex by studying the acquisition of TSPs without disrupting the complex’s functionality. Using kuttervirus S117 as a backbone, we demonstrated that acquiring *tsp* genes from *Kuttervirus* and *Agtrevirus* phages within the *Ackermannviridae* family led to altered host recognition. A fifth TSP was designed to interact with the branched complex and expand host recognition even further. Interestingly, the acquisition of *tsp5* resulted in a recombination event between *tsp4* and *tsp5* or deletion of *tsp3* and truncation of *tsp4* genes. Our study provides insight into the development of the branched TSP complex, enabling *Ackermannviridae* phages to adapt to different hosts.

## Highlights

• A branched complex of multiple tail spikes allows *Ackermannviridae* receptor binding
• Replacement of individual tail spikes did not disrupt the complex functionality
• Attempts to extend the complex with one more tail spike led to genetic changes
• The branched tail spike complex promotes adaptive host recognition

## Graphical abstract

**Figure:**
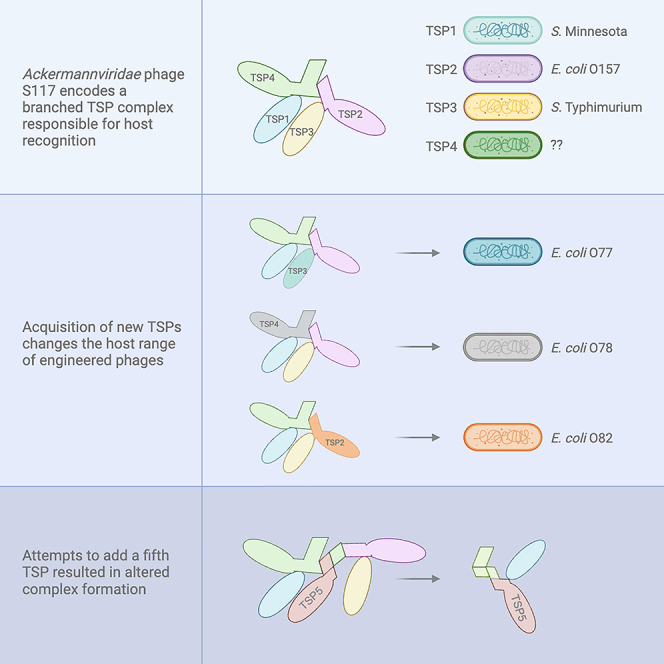

## Introduction

Bacteriophages (phages) encode receptor-binding proteins that form long or short tail fibers (TFs) or tail spike proteins (TSPs) attached as distal structures to the phage tail. When infecting a bacterial host, the phage receptor-binding proteins recognize the surface receptor of the bacterial host. The specificity of this initial phage-host interaction ensures the infection of proper hosts, and receptors have been suggested as the major determinant of the host range of phages.^1^ On the phage side, most characterized phages typically use a single receptor-binding protein to recognize their bacterial host. Yet, the rise of whole-genome sequencing has identified an increasing number of phages that encode multiple receptor-binding proteins. For example, gamaleyavirus phage G7C encodes two TSPs interacting in a complex where one of the TSPs deacetylates the O-antigen on *Escherichia coli*.^2^ Even more complex is the network of receptor-binding proteins of recently characterized phages; for instance, phage ϕKp24 encodes 14 TSPs, each proposed to recognize a distinct K-antigen of *Klebsiella*, including K2, K1, K25, K35, and KN4.^3^

The multiple receptor-binding proteins of these phages often form a branched complex by interacting through defined structural domains or entire proteins conserved even across phage families.^4^^,^^5^ For example, TSPs of *Klebsiella* phages carrying a branched TSP complex share similarities to domains 2 and 3 found in Gp10 of the T4 phage.^3^^,^^4^ In phage T4, Gp10 is part of the peripheral baseplate and consists of four domains that form an X shape and are crucial for assembling the tail fiber complex. Domains 2 and 3 of Gp10 adopt a conserved β-jellyroll fold interacting with the short tail fiber and the baseplate protein Gp9, which further interacts with the long tail fiber.^6^^,^^7^ Therefore, the ability to mediate protein-protein interactions may explain why Gp10-like domains are widespread in phages encoding multiple receptor-binding proteins arranged in a branched complex.

Phages of the *Ackermannviridae* family are another example of multiple TSPs linked together in a branched complex using Gp10-like domains. In these phages, Gp10-like domains are found as N-terminal conserved domains named XD.^8^ Most *Ackermannviridae* phages encode up to four different TSPs (hereafter referred to as TSP1 to TSP4). Interestingly, only two of them, TSP4 and TSP2, contain the conserved XD domains necessary for linking the four TSPs into a complex. Both TSP2 and TSP4 encode an XD2 domain that interacts to form the branched complex. Furthermore, TSP4 and TSP2 also encode an XD3 domain interacting with TSP1 and TSP3, respectively. Finally, all four TSPs contain tandem repeat domains (TDs), where the TD1 domain forms the N-terminal structural domain in TSP1 and TSP3. The role of the TD1 domains is to interact with the XD3 domains of TSP2 and TSP4. Lastly, the TSP4 contains an anchor domain in the N-terminal that interacts with the virion. To assemble the complex, TSP4 can interact with TSP1 before or after the interaction with TSP2. In contrast, TSP3 can only interact with the XD3 domain of TSP2 following the formation of the TSP4-TSP2 complex.^8^^,^^9^ Taken together, the four TSPs of *Ackermannviridae* phages encode several conserved N-terminal domains essential to establishg the branched TSP complex.

The host recognition of *Ackermannviridae* phages is mediated by the variable C-terminal of the four TSPs. This domain recognizes polysaccharides as receptors like lipopolysaccharide (LPS), exopolysaccharides, and capsular polysaccharides (CPSs), allowing the phages to infect diverse bacteria belonging to the *Enterobacteriaceae* family.^8^^,^^10^^,^^11^^,^^12^^,^^13^^,^^14^ We previously analyzed the TSP diversity in the *Kuttervirus*, *Agtrevirus*, *Limestonevirus*, and *Taipeivirus* genera of the *Ackermannviridae* family.^12^^,^^15^ Interestingly, a large pool of genetic diversity of receptor-binding domains was revealed, suggesting that phage receptor-binding proteins have been diversified to match the multitude of variations of O-antigen and K-antigens expressed by *Enterobacteriaceae*.^8^^,^^10^^,^^12^^,^^13^ While genetic variation of TSP receptor-binding domains has been demonstrated, especially in the *Kuttervirus* genus, it was also observed that many of the phages encode similar TSPs.^12^ For instance, 53 of the 69 *Kuttervirus* phages analyzed encode a similar TSP3 that binds to *Salmonella enterica subspecies* expressing either the O4 or O9 O-antigens.^12^ In contrast, phages in the *Agtrevirus* genus encode unique receptor-binding proteins, meaning that only a few phages share similar TSPs.^15^ However, the high degree of conservation of the XD and TD domains may serve as sites for homologous recombination and allow for acquiring new TSPs and diversifying the branched complex of *Ackermannviridae* phages. However, this remains to be experimentally proven.

This study aims to mimic the evolution of the branched TSP complex of *Ackermannviridae* phages by investigating the acquisition of novel TSPs and their impact on the assembly and functionality of the complex. We used kuttervirus phage S117 as a model phage and showed that this phage can functionally acquire entire *tsp* genes originating from phages belonging to the *Kuttervirus* and *Agtrevirus* genera of the *Ackermannviridae* family. To expand the branched TSP complex, we designed a TSP5 construct containing N-terminal domains interacting with the complex and the C-terminal of kuttervirus phage Det7, recognizing a novel host. Interestingly, this led to recombination between *tsp5* and *tsp4*, or deletion of *tsp3* and the truncation of *tsp4*. Our results give insight into the branched TSP network, allowing *Ackermannviridae* phages to adapt to new hosts.

## Results

### Conservation of the N-terminal TSP domains allows phage S117 to acquire entire *tsp* genes from another kuttervirus phage

**Figure 1:**
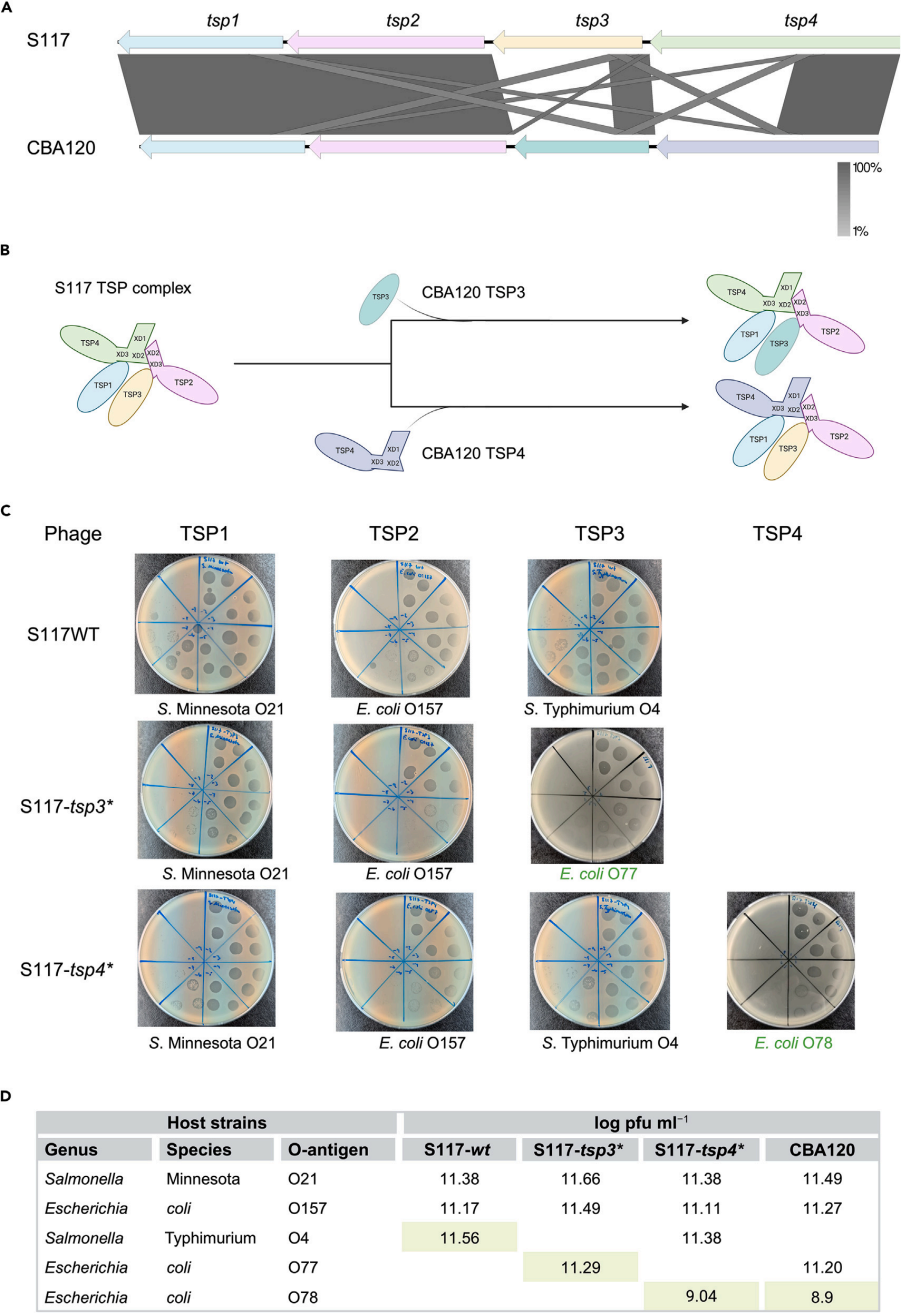
Phage S117 acquisition of *tsp3* and *tsp4* from *Kuttervirus* phage CBA120 (A) Comparison of the *tsp* locus of phages CBA120 and S117 demonstrate that the phages encode similar *tsp1* and *tsp2* genes, whereas the *tsp3* and *tsp4* genes only share similarity in the 5′ end. (B) Overview of the branched TSP complex of S117 before and after the acquisition of *tsp* genes from *Kuttervirus* CBA120. (C) Host range analysis of S117-*wt* and recombinant phages S117-*tsp3*∗ and S117-*tsp4*∗. The S117-*tsp3∗* phage infects the new TSP3 host (*E. coli* O77) and the TSP1 and TSP2 hosts of S117. Phage S117-*tsp4∗* infects the new TSP4 host (*E. coli* O78) and the TSP1, TSP2, and TSP3 hosts of S117. (D) Titer of S117-*wt*, the recombinant S117-*tsp3*∗ and S117-*tsp4*∗, and CBA120. The propagation strain is indicated by light green.

The structural N-termini domains of the TSPs in *Ackermannviridae* phages are essential for assembly and, consequently, functionality of the TSP complex. At the same time, the conserved sequence similarity in the 5′ end of the *tsp* genes may allow for homologous recombination between *tsp* genes, thus altering host recognition.^8^^,^^9^^,^^12^ To investigate the exchange of *tsp* genes between phages within the same genus, we used kuttervirus S117 as a model phage for acquiring novel *tsp* genes, whereas kuttervirus CBA120 was a source of such *tsp* genes. Analyzing the *tsp* gene cluster of phages S117 and CBA120 showed that *tsp1* and *tsp2* are highly similar (93.25% and 99.02% identity, respectively), whereas *tsp3* and *tsp4* only show similarity in the 5′ end of the genes (Figure 1A). For TSP3, the sequence similarity corresponds to the TD1 and TD2 domains, whereas the anchor domain, the three XD domains, and the TD1 and TD2 of TSP4 are conserved between the two phages (Figures S1 and S2). Therefore, due to the conservation of these important N-termini domains, we hypothesized that phage S117 could acquire the entire *tsp3* or *tsp4* genes of phage CBA120 and still maintain the integrity of the TSP complex (Figure 1B). Since the C-terminal receptor-binding domains of newly acquired TSPs are different, the functionality of the TSP complex can be verified by determining the host range of the recombinant phages. For this, we used previous data showing that TSP1 and TSP3 allow S117 to infect *Salmonella enterica* subspecies Minnesota O21 and Typhimurium O4 O-antigens, respectively, whereas TSP2 binds to the O157 O-antigen of *E. coli.* The host receptor for TSP4 has yet to be identified.^12^ Similarly, TSP3 and TSP4 of phage CBA120 allow infection of *E. coli* O77 or *E. coli* O78, respectively (Plattner et al.^8^).

To allow phage S117 to acquire *tsp3* or *tsp4* gene from phage CBA120, we cloned each of the *tsp3* and *tsp4* genes on a plasmid flanked with 500 bp sequences homologous to phage S117 and used a CRISPR-Cas9 system as counter selection. Homologous recombination was promoted by infecting *Salmonella* Typhimurium (LT2c ΔStyLTI), carrying the homologous template and the CRISPR-Cas9 system with phage S117. *Salmonella* Typhimurium LT2c, previously deleted for the StyLTI restriction-modification system, was used to maintain the two plasmids in the host.^16^ Subsequently, single plaques were picked and screened by PCR for the presence of the *tsp3* or *tsp4* genes of CBA120, respectively. Recombinant phages S117-*tsp3∗* and S117-*tsp4∗* were then isolated and shown to form plaques on the new hosts, *E. coli* O77 or *E. coli* O78, respectively. We observe a low titer when spotting on the *E. coli* O78 host when comparing to the other host. However, we observed similar low titer when spotting CBA120 on the native host, suggesting that it is not the *tsp4* gene recombination that is the cause for lower infection. In addition, we confirmed that the TSP complex was still functional by showing that the recombinant phages S117-*tsp3∗* and S117-*tsp4∗* could still form plaques on the remaining phage S117 hosts with an efficiency of plating similar to the wild-type S117 phage (Figures 1C and 1D). Furthermore, sequencing the recombinant phages S117-*tsp3∗* and S117-*tsp4∗* confirmed the exchange of *tsp* genes. Overall, we showed that phage S117 can acquire entire *tsp3* and *tsp4* genes from a phage within the genus and still preserve the functionality of the TSP complex.

### Phage S117 can acquire a new *tsp2* gene from agtrevirus phage AV101

**Figure 2:**
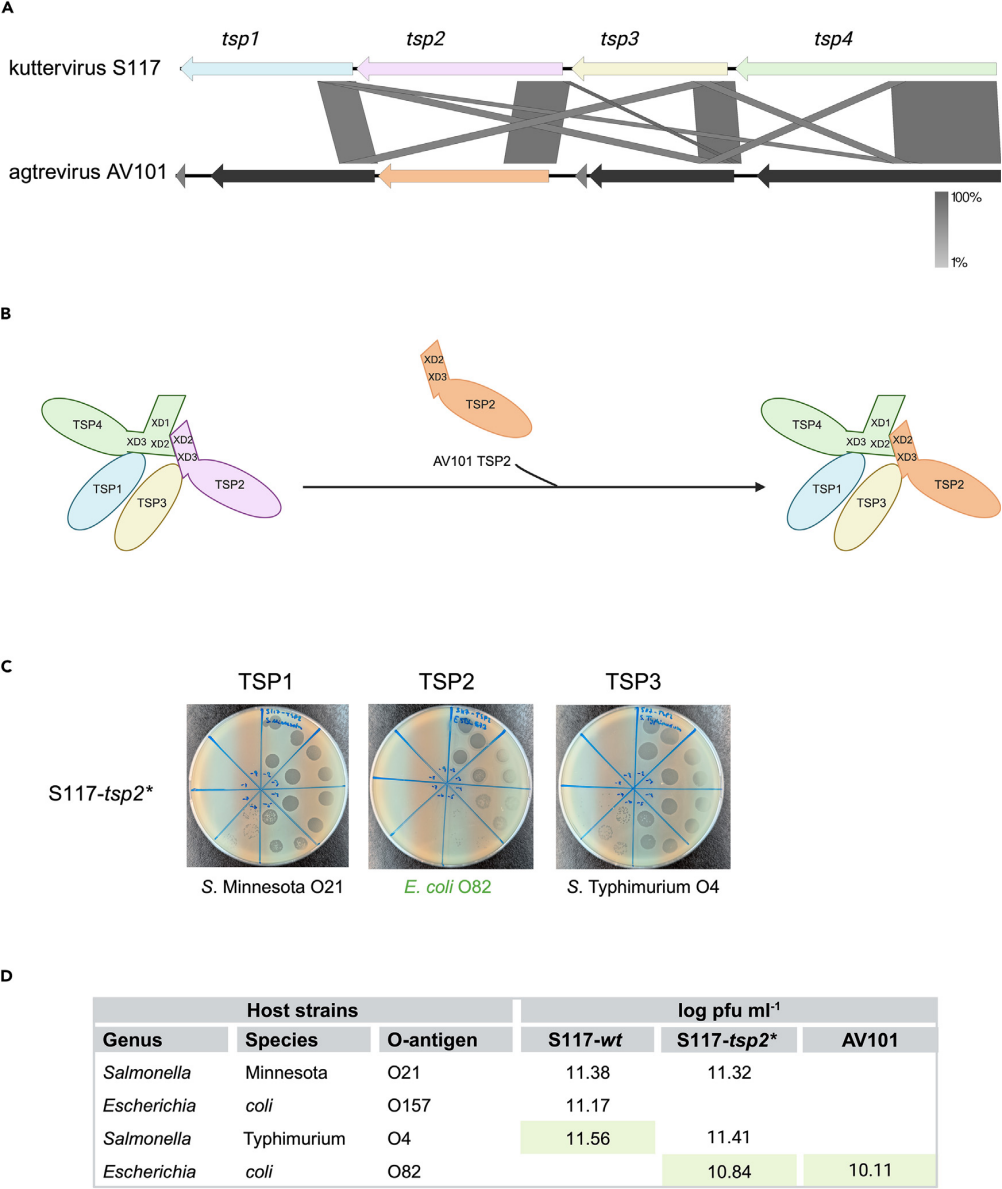
Phage S117 acquisition of *tsp2* from *Agtrevirus* phage AV101 (A) Comparison of the *tsp* locus of phages AV101 and S117 showed that all genes are similar in the 5′ end, and only other short regions are similar between the *tsp1*, *tsp3*, and *tsp4* genes of the two phages. (B) Overview of the branched TSP complex of S117 before and after the acquisition of *tsp2* from *Agtrevirus* phage AV101. (C) Host range analysis of recombinant phage S117-*tsp2*∗. The S117-*tsp2∗* phage infects the new TSP2 host (*E. coli* O82) and the TSP1 and TSP3 hosts of S117. (D) Titer of S117-*wt*, the recombinant S117-*tsp2*∗, and AV101. The propagation strain is indicated by light green.

Our previous analysis of the diversity of TSPs of *Ackermannviridae* phages revealed that similar TSPs were associated with phage genera, suggesting that exchange only occurs between phages in the same genus.^12^ However, we have recently shown that the receptor-binding domain of TSP4 of agtrevirus AV101 shared similarity with kuttervirus LPST94. As a consequence, an exchange may be possible between different genera within this family.^15^ Indeed, the 5′ end of *tsp* genes is highly similar, including the sequence encoding for the conserved XD domains of TSP2 and TSP4 and the TD1 domains of TSP1 and TSP3. We, therefore, speculated if *tsp* genes of agtrevirus AV101 belonging to another genus in the *Ackermannviridae* family could be exchanged by kuttervirus phage S117 and still form a functional TSP complex. Agtrevirus phage AV101 infects extended spectrum β-lactamaseproducing *E. coli* strains, and we previously showed that the four TSPs recognize *E. coli* O8, O82, O153, and O159 O-antigens, respectively.^15^ Alignment of the *tsp* gene clusters of phages AV101 and S117 shows sequence similarity in the N-termini conserved domains in all four TSPs (Figure 2). For example, the XD2, XD3, and TD1 domains of TSP2 are conserved (Figure S3), suggesting that TSP2 of AV101 can be incorporated into the S117 TSP complex. To demonstrate the functionality of this recombinant TSP complex, we used the same approach described earlier. Still, instead of screening for the new *tsp2* gene with PCR, we directly selected for plaque formation on the new host, *E. coli* O82. The recombinant S117-*tsp2∗* phage did indeed infect the new *E. coli* O82 host of AV101 TSP2 as well as the hosts of TSP1 and TSP3 of S117, *Salmonella* Minnesota O21 and *E. coli* O157, respectively (Figures 2B and 2C). Furthermore, whole-genome sequencing of the recombinant phage confirmed the exchange of the *tsp2* gene. In conclusion, these results demonstrate that a *tsp2* gene originating from *Agtrevirus* within the *Ackermannviridae* family can be exchanged across genera and still produce infectious phages.

### An attempt to add a *tsp5* gene led to recombination between *tsp4* and *tsp5*

**Figure 3:**
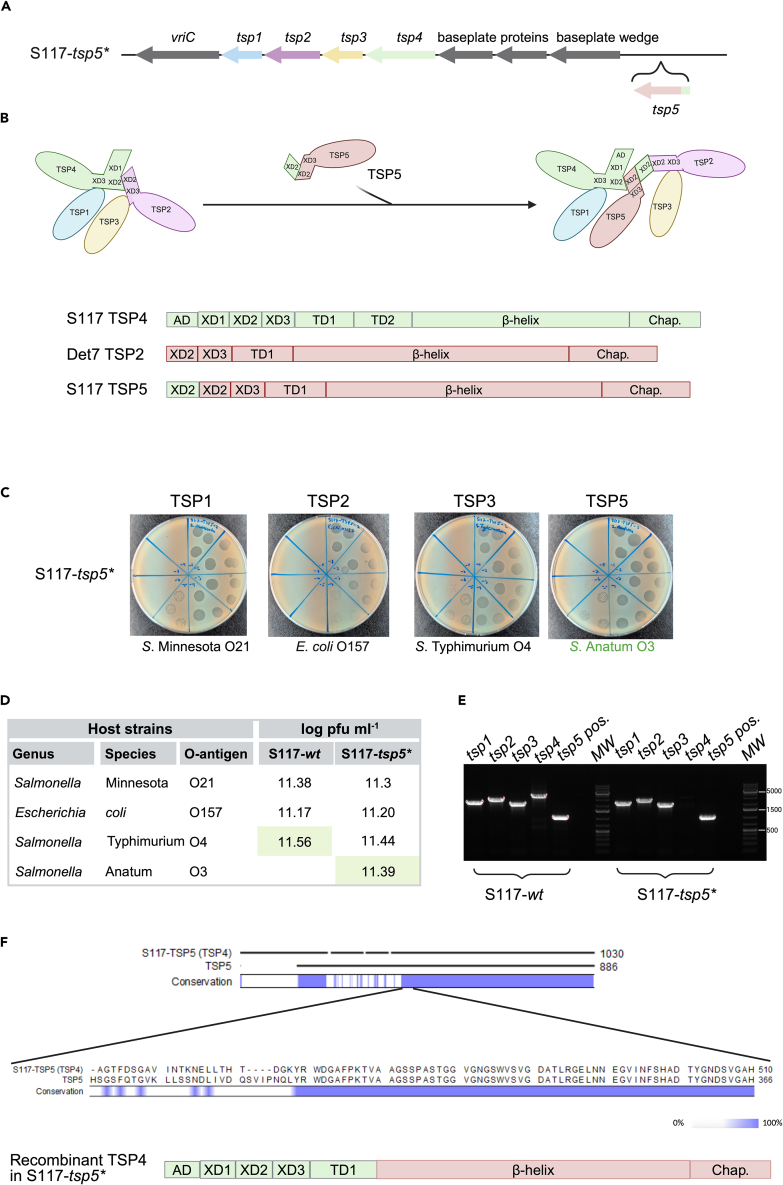
Selection for phage S117-*tsp5∗* results in recombination between S117 *tsp4* and *tsp5* genes (A) Location of the additional *tsp5* gene in the genome of phage S117. (B) Overview of the designing of the synthetic *tsp5* gene composed of the XD2 domain of *tsp4* of phage S117 fused to the entire *tsp2* gene of phage Det7 and model for the expanded TSP complex. (C) Host range analysis of recombinant phage S117-*tsp5*∗. The S117-*tsp5∗* phage infects the new TSP5 host (*S*. Anatum) and the TSP1, TSP2, and TSP3 hosts of S117. (D) Titer of S117-*wt* and the recombinant S117-*tsp5*∗. The propagation strain is indicated by light green. (E) PCR using primers as indicated in materials and methods to detect the four *tsp* genes and determine the location of the *tsp5* genes. Ladder: GeneRuler 1 kb plus. (F) Whole-genome sequencing of recombinant S117-*tsp5*∗ demonstrates a recombination event between *tsp4* and *tsp5*, resulting in a chimeric TSP4 protein containing the N-terminal of TSP4 and the receptor-binding domain of the TSP5.

The XD domains in TSP2 and TSP4 are crucial for assembling the branched TSP complex since the XD2 domains of TSP2 and TSP4 interact (Figure 3B).^8^^,^^9^ We speculated if an additional TSP (TSP5) could be incorporated into the complex by carrying an XD2 domain, for example by adding this to an existing TSP2, as we reasoned that a TSP2 containing two XD2 domains could interact with both TSP4 and TSP2 (Figure 3B). To create such a novel *tsp5*, we added the XD2 domain of *tsp4* of S117 upstream of the entire *tsp2* gene originating from *kuttervirus* Det7 (recognizing the O3 O-antigen from *Salmonella* Anatum).^17^ A schematic representation of the synthetical construct is presented in Figure S4. To prevent disrupting the transcription of the remaining *tsp* genes, we aimed to insert the novel *tsp5* downstream of the *tsp* gene cluster following the successful strategy described earlier (Figure 3A). To isolate recombinant phages, we utilized the same methods as before and spotted the stock containing phages with potential recombinant genomes directly on the new *S.* Anatum host and isolated plaques representing S117 carrying TSP5. Indeed, a recombinant phage S117-*tsp5∗* was able to infect the new *S.* Anatum host and the hosts recognized by TSP1, TSP2, and TSP3 (Figures 3C and 3D). As we do not know the receptor of TSP4, and hence the host of S117, we verified by PCR that all *tsp* genes, including *tsp5*, were present in the recombinant phage S117-*tsp5*∗. Our results showed that amplification of the region for the *tsp5* gene insert did not correspond to the expected size (3658 bp) but was identical to the wild type S117 (1,000 bp) (Figure 3E). In addition, the *tsp4* gene of the recombinant S117-*tsp5∗* genome could not amplify (Figure 3E). We hypothesized that a recombinant event between *tsp4* and *tsp5* may have created a phage able to infect the new host. To investigate this further, we sequence-verified the genome of phage S117-*tsp5∗.* We found that the receptor-binding domain of *tsp4* was replaced by the receptor-binding domain of *tsp5*, indeed suggesting recombination between *tsp4* and *tsp5* (Figure 3F). Furthermore, we did not observe any mutations or SNP in the recombinant genomes. Overall, we did not manage to introduce an additional *tsp* gene into the genome of S117 that could interact with the TSP complex. Instead, the recombination event created a phage with a *tsp4* containing the novel receptor-binding domain of phage Det7, allowing the phage to infect *S*. Anatum.

### Acquisition of a *tsp5* gene resulted in the deletion of *tsp3* and truncation of *tsp4*

**Figure 4:**
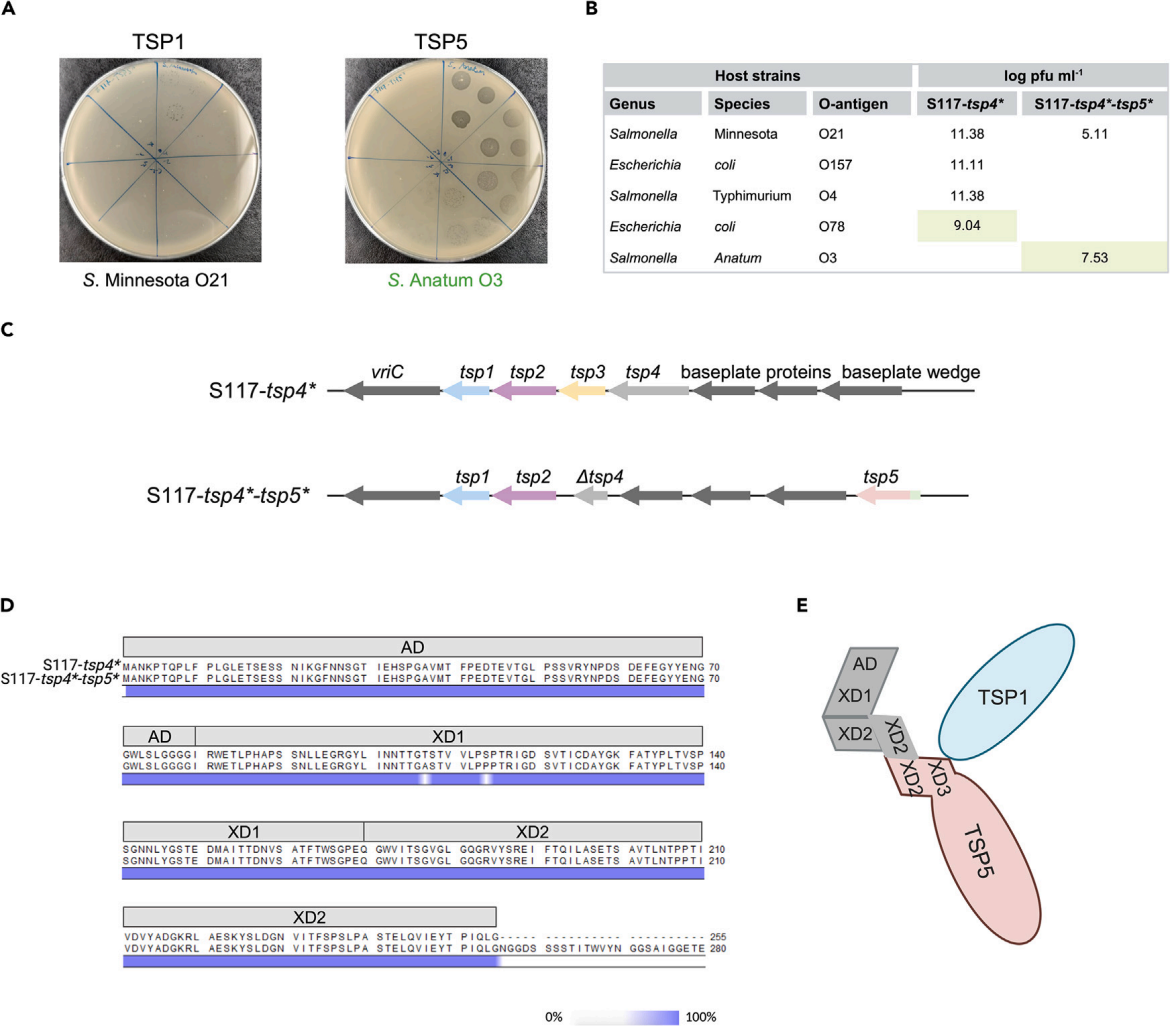
Acquisition of *tsp5* leads to the deletion of *tsp3* and most of *tsp4* (A) Host range analysis of recombinant phage S117-*tsp4*∗-*tsp5*∗. The new modified S117-*tsp4∗*-*tsp5*∗ phage infects the host of TSP5 (*S.* Anatum). (B) Recombinant phage S117-*tsp4∗*-*tsp5*∗ infects *S.* Anatum with a higher titer compared to the TSP1 host *S*. Minnesota and showed no infection of TSP2, TSP3, and TSP4 hosts. The propagation strain is indicated by light green. (C) Whole-genome sequencing of phage S117-*tsp4∗*-*tsp5*∗ revealed a deletion of *tsp3* and most of the *tsp4* gene. (D) Sequence alignment of the N-terminal domains of TSP4 of S117-*wt* and the TSP4 of S117-*tsp4∗*-*tsp5*∗ showed high similarity until the XD2 domain. Only the XD domains necessary for interactions within the TSP network were conserved in the S117-*tsp4∗*-*tsp5*∗ phage. (E) Proposed TSP complex of recombinant phage S117-*tsp4∗*-*tsp5*∗.

In a second attempt to create S117 phages carrying an additional *tsp5* gene, we used the previously isolated recombinant phage S117-*tsp4∗* encoding TSP4 from CBA120 instead of wild type S117, thus allowing for screening for all five TSP hosts (Figure 3). We indeed isolated a recombinant phage (S117-*tsp4∗*-*tsp5∗*) that infects *S.* Anatum (Figure 4A). Unexpectedly, phage S117-*tsp4∗*-*tsp5∗* could not infect hosts recognized by TSP2, TSP3, and TSP4 and could barely infect the TSP1 host (Figures 4A and 4B). We hypothesized that the results could suggest that the additional XD2 domain of *tsp5* may disturb the assembly of the TSP complex. However, while sequencing of the *tsp* cluster demonstrated that *tsp5* was correctly inserted into the genome, no reads mapped to the *tsp3* gene and the 3′ end of the *tsp4* gene, while the recombinant phage genome could not be fully closed (Figure 4C). Yet, further analysis confirmed that the N-terminal sequences encoding the anchor domain and the XD1 and XD2 domains of the *tsp4* gene were present in our sequencing data (Figure 4D). Combining the sequencing data and host range results suggests that S117-*tsp4∗*-*tsp5∗* only carries the functional receptor-binding domains of TSP1 and TSP5 (Figure 4E). Overall, the acquisition of a *tsp5* into the genome resulted in the deletion of *tsp3* and truncation of *tsp4* but still encoded the TSP4 domains required for assembly and functionality of the TSP complex.

## Discussion

TSPs allow phages to successfully infect their host by recognizing specific polysaccharide bacterial receptors like the LPS or CPS.^8^^,^^11^^,^^14^^,^^18^ However, bacteria express a high diversity of these polysaccharides within a species.^19^^,^^20^^,^^21^ For instance, *E. coli* isolates combined display an array of 185 different O-antigens of LPS.^20^ Therefore, phages encoding TSPs may be limited in their host range compared to phages that recognize a protein receptor of a more conserved nature within a bacterial species. For instance, the receptor-binding protein of gelderlandvirus phage S16 recognizes the outer membrane protein OmpC as the receptor, and due to the conserved nature of the protein, the phage can infect a broad range of *Salmonella enterica* serotypes.^22^ To allow infection of a broader selection of hosts, phages using TSPs as receptor-binding proteins have evolved different mechanisms to adapt to the large diversity of polysaccharide receptors. Here, we studied *Ackermannviridae* phages encoding four TSPs forming a branched complex protruding the baseplate. We aimed to mimic diverse mechanisms that allow these phages to acquire novel TSPs and investigated the impact of novel TSPs on the assembly and functionality of the complex.

Recent research has proposed that bacterial receptors are the major determinant of phage susceptibility, being even more important than internal phage resistance mechanisms.^1^ Hence, phages have evolved different strategies to overcome the diversity of phage receptors. One approach to adapting to a new host is to exchange receptor-binding domains between phages through homologous recombination.^23^^,^^24^^,^^25^^,^^26^ Indeed, *in silico* analysis of *tsp* genes in the *Ackermannviridae* family has suggested that *tsp* genes undergo homologous recombination due to the conserved 5′ end of the four genes.^12^ Our study showed that homologous recombination can exchange *tsp* genes and still produce infectious phages without disrupting the TSP complex, similar to a recent study.^27^ While we showed exchange within the family, other *in-silico* analyses of TSPs in the *Ackermannviridae* family have demonstrated that the receptor-binding domains are exchanged with distantly related lytic phages and prophages.^11^^,^^12^^,^^15^ For instance, the receptor-binding domain of TSP4 of phage AV101 is similar to kayfunavirus ST31 and phapecoctavirus Ro121c4YLVW, both distantly related phages.^15^ While domain exchange seems to be prevalent in the family, the frequency of exchange is not known. Co-evolution studies of bacteria and phages often show that phages adapt to new hosts by point mutations in the receptor-binding domain and not through domain exchange.^28^^,^^29^ This may be due to the limited number of bacteria and phages in a given co-evolution experiment, thereby limiting the natural complexity. Thus, while we observe and show that exchanging whole genes or domains is a possible strategy for adaption to new hosts, the exchange frequency is unknown and remains to be determined.

While the exchange of genes of domains of receptor-binding proteins is one way of adapting to a new environment, phages have also evolved to express multiple receptor-binding proteins.^2^^,^^3^^,^^4^^,^^30^ Phages in the *Stephanstirmvirinae* subfamily, e.g., phage phi92, encode up to five receptor-binding proteins including both TSPs and TFs each protruding the baseplate.^31^^,^^32^ Other phages encode proteins or domains that anchor the receptor-binding proteins, like the Gp10-like domains in *Ackermannviridae* phages, to form a complex.^4^^,^^8^^,^^9^ In *Ackermannviridae* phages, it was previously suggested that the phages have evolved by gene duplication from a single TSP (TSP4) and that acquisition of the Gp10-like domains allowed multiple TSPs to interact in a complex with TSP4.^8^^,^^12^ In most *Limestonevirus* phages, the TSP4 does not carry a receptor-binding domain but only the N-terminal structural domains necessary for assembling the TSP complex, including the remaining TSPs. Furthermore, *Limestonevirus* phages do not encode a *tsp3* gene.^33^^,^^34^^,^^35^ Similarly, we observed that attempting to introduce a fifth TSP resulted in a recombinant phage without a TSP3 and a TSP4 with only the N-termini structural domains. Thus, TSP4 may have been an adaptor protein that established the ability to form a complex and gained a receptor-binding domain through evolution. While we did not successfully add a fifth *tsp* gene in the genome of S117, two recent studies suggested that *Taipeivirus* phages like KpS110 and Menlow encode five TSPs.^4^^,^^36^ Bioinformatic analysis of these phages showed that all five genes in the *tsp* cluster encode proteins that adopt a β helix fold common for all TSPs. Neither of the studies investigated the N-termini structural domains of the fifth TSP nor showed if the TSP is incorporated in the TSP complex. Still, overall, it may suggest that it is, in fact, possible to add a fifth TSP into the complex. In our TSP5 design, we incorporated an XD2 domain into a *tsp4* gene of Det7. The XD domains of TSP5 showed a shared sequence similarity with the XD domains present in TSP2 and TSP4 of S117. This overall sequence similarity could be the reason behind the observed different recombination events. Overall, our results also demonstrate that it is possible to engineer phages with multiple TSPs and could be used in further studies with complexes that carry Gp10-like domains.

### Limitations of the study

In our study, we investigated the potential of introducing a fifth *tsp* gene into the genome of S117. Despite multiple attempts, we were unsuccessful in obtaining the planned engineered phage. Instead, we observed various recombination events that modified the existing *tsp* genes within the genome. To minimize the likelihood of recombination events affecting other tsp genes, we propose a different design for the *tsp5* gene, ensuring that the XD domains do not share nucleotide sequence similarity with the native *tsp* genes in the genome. It is worth noting that our study employed a CRISPR-Cas9 system as a counter-selection method, which does not accurately replicate *in vivo* conditions. To simulate a true recombination event, co-infection of two *Ackermannviridae* phages would be necessary.

## Resource availability

### Lead contact

Further information and requests for resources and reagents should be directed to and will be fulfilled by the lead contact, Lone Brøndsted (lobr@sund.ku.dk).

### Materials availability

Bacterial isolates and bacteriophages are available by request from the lead contact under the conditions of a material transfer agreement (MTA).

### Data and code availability

• Reads and maps of recombinant bacteriophage genomes are available upon request from the lead contact. For accession numbers for phage genomes, see key resources table.
• This paper does not report original code.
• Any additional information required to re-analyze the data reported in this paper is available from the lead contact upon request.

## Acknowledgments

This project has received funding from the 10.13039/501100004836Independent Research Fund Denmark (9041-00159).

## Author contributions

A.N.S.: conceptualization, methodology, validation, formal analysis, investigation, writing – original draft, and writing – review and editing. D.K.: validation, formal analysis, and investigation. C.W.: conceptualization, methodology, and writing – review and editing. J.P.: formal analysis and writing – review and editing. R.L.: writing – review and editing and funding acquisition. M.C.H.S.: conceptualization, writing – review and editing, and funding acquisition. L.B.: conceptualization, project administration, supervision, visualization, writing – review and editing, and funding acquisition.

## Declaration of interests

The authors declare no competing interests.

## STAR★Methods

### Key resources table

**Table:** 

REAGENT or RESOURCE	SOURCE	IDENTIFIER
**Bacterial and virus strains**		

Stellar™ chemical competent cells	Takara Bio	N/A
*S*. Typhimurium O4 (LT2c ΔStyLTI)	Woudstra et al.^16^	N/A
*S*. Anatum O3	Robert Koch Institute	N/A
*E. coli* O157	NCTC12900	N/A
*E. coli* O77	Plattner et al.^8^	N/A
*E. coli* O78	Plattner et al.^8^	N/A
*E. coli* O82	Vitt et al.^37^	N/A
*E. coli* O18a	Robert Koch Institute	N/A
Kuttervirus CBA120	Plattner et al.^8^	GenBank: JN593240.1
Agtrevirus AV101	Vitt et al.^38^	GenBank: OQ973471.1
Kuttervirus S117	Gencay et al.^39^	GenBank: MH370370.1
S117-*tsp2*∗	This study	N/A
S117-*tsp*3∗	This study	N/A
S117-*tsp4*∗	This study	N/A
S117-*tsp5*∗	This study	N/A
S117-*tsp4*∗-*tsp*5∗	This study	N/A

**Chemicals, peptides, and recombinant proteins**		

Lysogeny Broth	Oxid	Cat#CM1023
DNase I (1 U/μL)	Thermo Fisher Scientific	Cat#EN0521
RNase A (10 mg/mL)	Thermo Fisher Scientific	Cat#EN0531
Proteinase K 50 μg/mL	Thermo Fisher Scientific	Cat# EO0491
EDTA (pH 8)	Thermo Fisher Scientific	Cat#R1021
Glycogen	Thermo Fisher Scientific	Cat# R0551
Ammonium acetate	Sigma	Cat#A1542
Kanamycin	Sigma	Cat#BP906
Spectinomycin	Sigma	Cat# S4014
L-arabinose	Sigma	Cat#A3256
Water, nuclease-free	Thermo Fisher Scientific	Cat#R0582
pEcgRNA	Addgene	RRID:Addgene_166581
pEcCas	Addgene	RRID:Addgene_73227
pEcgRNA-guide-TSP1	This study	N/A
pEcgRNA-guide-TSP2	This study	N/A
pEcgRNA-guide-TSP3	This study	N/A
pEcgRNA-guide-TSP4	This study	N/A
pEcgRNA-guide1-TSP5	This study	N/A
pEcgRNA-guide2-TSP5-2	This study	N/A
pTwist-Det7-TSP	This study	Twist Bioscience

**Critical commercial assays**		

DNA Clean & Concentrator-25	Zymo Research	Cat#D4011
NEBNext Ultra DNA Library Prep kit	New England Biolabs	Cat#E7370L
GeneJET PCR Purification Kit	Thermo Fischer Scientific	Cat#K0702
DreamTaq Green PCR Master Mix	Thermo Fischer Scientific	Cat#K1081
CloneAmp HiFi PCR Premix	Takara Bio	Cat#639298
In-fusion® HD-cloning kit	Takara Bio	Cat#638947
Qubit dsDNA BR Assay Kit	Thermo Fisher Scientific	Cat# Q32850

**Oligonucleotides**		

CRISPR guides used for *tsp* exchange	See Table S1	N/A
Primers for *tsp* exchange	See Table S2	N/A

**Software and algorithms**		

CRISPR guide RNA tool	Benchling®	https://www.benchling.com
CLC Genomics Workbench 9.5.3	Qiagen	N/A
CLC Workbench 21	Qiagen	N/A
BLAST	NCBI	https://blast.ncbi.nlm.nih.gov/Blast.cgi
EasyFig	Sullivan et al. 2011^40^	http://mjsull.github.io/Easyfig/files.html

### Experimental model and study participant details

#### Bacterial strains and growth conditions

The bacterial strains used in this study are listed in key resources table. Strains were cultured in LB medium (Lysogeny Broth, Merck, Darmstadt, Germany) at 37 37°C and 170 rpm.

### Method details

#### Phage propagation

Kuttervirus phages S117, CBA120, and agtrevirus phage AV101 were propagated on the host *S.* Typhimurium (LT2c), *E. coli* (NCTC12900) and *E. coli* (ESBL040) strains, respectively, as described earlier.^12^ Single colonies of the propagating strains were inoculated into LB medium and incubated at 37°C shaking at 170 rpm until reaching the exponential phase (approximately 5 h). Previous stocks of the phages were diluted to 10^3^-10^5^ PFU/mL, and 330 μL of the phage dilutions and 330 μL of the hosts were mixed before 12 mL top agar (Lbov; LB broth with 0.6% Agar bacteriological no.1, Oxoid) were added to the mixture and poured onto three LA plates (LB with 1.2% agar). After the top agar was solidified, they were incubated overnight at 37°C. The next day, 5 mL SM buffer (0.1 M NaCl, 8 mM MgSO4·7H2O, 50 mM Tris-HCl, pH 7.5) was added to each of the three plates followed by incubation of the plates overnight at 4°C and shaken at 50 rpm. The SM buffer was collected and centrifuged at 11,000 rpm at 4°C for 15 min and filtered twice with 0.2 μM filters. The new phage stocks were stored at 4°C before use. The titer of the stocks was determined with a plaque assay (described below).

#### Phage host range analysis

To determine the host range of CBA120, AV101, and S117 phages, plaque assays were carried out.^39^ Briefly, single colonies of strains of interest were inoculated into a 5 mL medium and incubated at 37°C shaking at 180 rpm for approximately 5 h to reach the exponential phase. Subsequently, 100 μL of the strain was mixed with 4 mL of top agar and poured onto LA plates. The plates were dried, and 10-fold serial dilutions of the phage stocks were spotted onto the top agar plates. After overnight incubation, the plates were screened for single plaques, and the plating efficiency was calculated by comparing the PFU/mL of the tested strains with the PFU/mL of the propagation strain.

#### Phage DNA isolation

Phages DNAs were isolated before engineering or DNA sequencing.^12^ RNase A and DNase I(Thermo Fischer Scientific) were added to the sample with a final concentration of 10 μg/mL and 20 μg/mL, respectively, prior to the nucleic acid extraction. The sample mixture was incubated for 20 min at 37°C followed by the addition of sterile EDTA (pH 8) (Thermo Fischer Scientific) at a concentration of 20 mM. To degrade the phage capsid, 50 μg/mL of Proteinase K (Thermo Fischer Scientific) was added to the sample and incubated for 2 h at 56°C. The release of the DNA was verified on a 1% agarose gel. Following, the genomic DNA Clean & ConcentratorTM-10 kit (Zymo Research) was used to isolate the phage genome using the manufacturer’s instructions. The concentration of the isolated DNA was measured with Qubit (Thermo Fisher Scientific), and the quality of the DNA was verified on a 1% agarose gel.

#### Phage engineering

Genetic engineering of phages was performed through CRISPR/Cas9, as previously described.^16^ Briefly, we used a recently developed two plasmids system, pEcCas (addgene #73227) and pEcgRNA (addgene #166581), where Cas9 was encoded by the pEcCas plasmid, and the Cas9 RNA guide could be cloned into the pEcgRNA plasmid as well as the recombinant template used to modify the phage genomes. Furthermore, the pEcCas plasmid also expressed the Lambda Red system under an inducible arabinose promoter to increase recombination efficiency.^41^

#### Guide RNA efficiency

Guide RNA (gRNA) targeting the four S117 TSPs were designed using the CRISPR tool on the Benchling Website. A list of the efficient guides for each tsp gene is presented in Table S1. The guides were introduced into the pEcgRNA vector through reverse PCR using the CloneAmp HiFi PCR Premix (Takara Bio) following the manufacturer’s instructions. Primers for cloning the guides into pEcgRNA are presented in the Appendix (Table S2). The PCR products were purified using Zymo PCR purification kit (Zymo Research) and directly transformed into Stellar chemical competent cells (Takara Bio) and plated on spectinomycin (50 μg/mL) plates. The pEcgRNA-guide plasmids from an overnight culture of Stellar cells expressing the plasmids grown in LB medium with spectinomycin (50 μg/mL) were extracted using GeneJET Plasmid Miniprep Kit (Thermo Scientific). The pEcgRNA-guide plasmids were individually transformed into LT2c ΔStyLTI competent cells harboring the pEcCas plasmid and plated on both spectinomycin for selection of pEcgRNA (50 μg/mL) and kanamycin for selection of pEcgRNA-guide plates (50 μg/mL). The next day, the presence of both plasmids was screened by colony PCR using DreamTaq Green PCR Master Mix (Thermo Fischer). Guide efficiency was evaluated by plaque assay on LT2c ΔStyLTI containing pEcgRNA-guide and pEcCas and on LT2c ΔStyLTI containing only pEcCas as a control (Figure S4).

#### Construction of the recombinant templates

The guides with the highest efficiency were further used for *tsp* gene exchange. All primers used are presented in Table S2. To exchange the *tsp* genes in *Kuttervirus* S117, we constructed a recombinant template (RT). More specifically, the *tsp* gene and 500 base pair upstream and downstream of the S117 *tsp* gene that should be exchanged (Left and Right Homology Arms (LHA and RHA)) were amplified by PCR using CloneAmp HiFi PCR Premix (Takara Bio). LHA and RHA primers carried overhangs with homology to the *tsp* exchanged gene. After PCR purification, the RT was constructed by Splicing by Overhang Extension (SOE) PCR. Briefly, the LHA and RHA PCR products were used as primers for amplifying the *tsp* exchange gene using CloneAmp HiFi PCR Premix (Takara Bio). After five PCR cycles, primers amplifying the whole new construct were added, and the PCR was continued for 30 additional cycles. To verify the correct assembly of the three fragments 5 μL of the PCR reaction was run on a 1% agarose gel. Correct amplicons were isolated by Zymo PCR purification kit (Zymo Research). The PCR products were cloned into a PCR linearized pEcgRNA-guide plasmid to make the pEcgRNA-guide-RT plasmid using In-fusion HD-cloning kit (Takara Bio) following the manufacturer’s instructions.

#### Exchange of *tsp* genes

The LT2c expressing both pEcCas and the different pEcgRNA-guide-RT plasmids were grown at 37°C to an OD600 = 0.2 before 0.1% arabinose was added to induce the expression of the Lambda Red system. The strains were further grown at 37°C for 2 h followed by a plaque assay to perform the recombination between the phage and the pEcgRNA-guide-RT plasmid. The next day, plaques were screened for the presence of the new *tsp* genes with DreamTaq Green PCR Master Mix (Thermo Fischer). Positive plaques were then used for a new plaque assay with the new host to test if the recombinant phage could infect the host. The recombinant phages were further propagated on the new host.

#### Construction of TSP5 engineered phage

To construct a phage with an additional *tsp* gene (*tsp5*) we designed a *tsp2* gene from kuttervirus phage Det7 (accession number NC_027119) with an additional XD2 domain from S117 *tsp4* gene in the start of the gene (Figure S5). Furthermore, the promoter for the *tsp2* of Det7 was added to the construct. The designed gene was ordered from Twist Bioscience. To insert the *tsp5* gene into the genome of S117 we utilized the same method as for exchanging the *tsp* genes (see phage engineering section). Guide RNAs are presented in Table S1 and primers for cloning into pEcgRNA are presented in Table S2.

#### Phage DNA sequencing

Using a Qubit 2.0 instrument and the Qubit dsDNA BR Assay Kit (Thermo Fisher Scientific), the quality of the DNA preparations of genomes of S117 and recombinant S117 was evaluated. The NEBNext Ultra DNA Library Prep kit from New England Biolabs was used to prepare the genomes for Illumina sequencing, and the HiSeq 4000 machine (Illumina) was used to sequence the samples. The CLC Genomics Workbench 21 (Qiagen, Aarhus) with default settings was used to assemble the raw reads after trimming.

### Quantification and statistical analysis

#### Bioinformatic analysis

The genomes of *Kuttervirus* phages S117 (accession number MH370370) and CBA120 (NC_016570), and *Agtrevirus* phage AV101(OQ973471) were used for analysis of their *tsp* gene cluster and for designing primers for *tsp* exchange. Sequence similarities of the *tsp* gene clusters were visualized using EasyFig version 2.2.5 with default settings (BLAST options, min. length 0, max. e-value 0.001, min. identity value 0.). Alignments of individual *tsp* genes of CBA120, AV101, and S117 were also done in CLC Workbench 22 with default settings (Gap open cost = 10,0, Gap extension cost = 1,0, End gap cost = As any other, Alignment mode = Very accurate (slow), Redo alignments = No, Use fixpoints = No).
